# Inter-subject variability of pleasant pain relief using a data-driven approach in healthy volunteers

**DOI:** 10.3389/fpain.2022.1003237

**Published:** 2022-11-21

**Authors:** Catherine Henri, Serge Marchand, Charles-Édouard Giguère, Guillaume Léonard, Stéphane Potvin

**Affiliations:** ^1^ Research Center of the Montreal Mental Health University Institute, Montreal, QC, Canada; ^2^ Department of Psychiatry and Addictology, University of Montreal, Montreal, QC, Canada; ^3^ Department of Neurosurgery, Faculty of Medicine and Health Sciences, University of Sherbrooke, Sherbrooke, QC, Canada; ^4^ Research Centre on Aging, School of Rehabilitation, Faculty of Medicine and Health Sciences, University of Sherbrooke, Sherbrooke, QC, Canada

**Keywords:** pleasant pain relief, conditioned pain modulation (CPM), inter-subject variability, temporal summation of pain (TSP), pain mechanisms, thermode, cold pressor test (CPT)

## Abstract

**Background:**

The offset of a painful and unpleasant sensation can elicit pleasure. This phenomenon, namely pleasant pain relief (PPR), is attracting growing interest in research. While the cold pressor test (CPT) has been frequently used to study the inhibition of pain by the administration of another painful stimulation (inhibitory conditioned pain modulation; ICPM), a preliminary study from our research team has shown that CPT can also elicit a robust and long-lasting PPR. However, its effects on pain relief and inhibition vary greatly between subjects. Although substantial research has been carried out on inter-individual variability in the case of ICPM, the same cannot be said of PPR. Therefore, the current study sought to identify clusters of healthy volunteers with similar dynamic pain responses during the CPT, using a data-driven approach, and to investigate the inter-subject variability for PPR and ICPM.

**Methods:**

One hundred and twenty-two healthy volunteers were recruited. A sequential ICPM paradigm was carried out with CPT (water at 10°C) and a Peltier Thermode to evaluate pain intensity and unpleasantness. Moreover, PPR was measured for four minutes at CPT offset. Statistical analyses were performed using group-based trajectory modelling.

**Results:**

Four trajectories (groups) were identified for CPT pain intensity and unpleasantness ratings with varying levels of tonic pain and pain sensitization (e.g., temporal summation). PPR scores were correlated with both pain ratings trajectories (*p* < 0.001). On the other hand, no differences were found between groups regarding ICPM efficacy (percentage pain inhibition).

**Discussion:**

This study has provided a first step into the investigation of PPR and ICPM interindividual variability. Using a data-driven approach, it was shown that PPR at CPT offset differs between clusters of participants identified based on dynamic pain intensity and unpleasantness responses from CPT. Thus, it was brought to light that both the levels of tonic pain and pain sensitization underlie individual differences in PPR. The lack of correlation between CPT pain trajectories and ICPM efficacy may be explained by the hypotheses that eliciting ICPM requires only a certain threshold of stimulation which doesn’t need to be noxious. In the future, studies on the inter-subject variability of PPR in large samples of chronic pain patients are warranted.

## Introduction

Pain is an unpleasant sensation that we tend to avoid. It has been defined as “a distressing experience associated with actual or potential tissue damage with sensory, emotional, cognitive, and social components.” (1). The natural motivation to avoid pain and seek reward is an essential survival mechanism as it prevents harm and injury. However, pain can also become chronic in which case it loses its original purpose of protective properties and has, too often, devastating consequences on those who suffer from it, on their relatives and on society (2–4). Chronic pain’s prevalence is very high as it has affected nearly 7.6 million Canadians in 2021 (5). In the United States, chronic pain prevalence is estimated to be between 18% and 34.5% (6). Chronic pain states are often bidirectionally related to comorbid conditions, like depression and anxiety (7). Although, alterations in pain modulation mechanisms in chronic stages are well described, much remains to be discovered about how pain offset affects its perception. Indeed, it is becoming increasingly clear that what happens when pain ends also plays an important role in its modulation (8, 9).

While pain modulation research has traditionally focused on central sensitization and inhibitory mechanisms such as conditioned pain modulation (CPM), research highlights complex links between pleasure and pain. Historically, studies have shown that the administration of pleasurable stimuli (music, smells, attractive faces, etc.) produces analgesic effects (10–12). More recently, a few research teams have observed that pain offset is often accompanied by a pleasant relief (8, 13, 14). This phenomenon has been explained by the opponent process theory which postulates that all deviations from homeostasis are accompanied by a process of the opposite valence (15–17). Thus, if a primary sensation (such as a painful one) is abruptly terminated, a sensation at the other side of the spectrum (e.g., pleasure) will be felt (14).. In the field of addiction, it has been observed that the long-term use of psychoactive substances is associated with dysphoria and painful somatic symptoms (18). In fact, the acute effects of drug consumption (e.g., euphoria, pleasure) are counteracted by opponent feelings as the drug wears off. With time, the withdrawal symptoms get worse, which further promotes drug-seeking behaviors to avoid the pain they create.

The cold pressor test (CPT) is well suited to evaluate the opponent process mechanisms. CPT, an experimental design widely used to study pain perception, involves immersing a subject’s limb in cold water for different lengths of time (19, 20). This test has been linked to the activation of the somatosensory cortex, implicated in pain intensity, and other regions like the amygdala, the insula and the anterior cingulate cortex that are mostly related to pain unpleasantness (21, 22). Furthermore, CPT induces the unpleasant experience of pain. Hence, it could be expected that removing this stimulus would generate a phenomenon of pleasant pain relief (PPR). Leknes et al. tested this phenomenon by delivering multiple painful stimuli with a thermode applied on the hand for three-second intervals, and found a significant PPR of five seconds (9). However, a preliminary study from our research team has shown that CPT elicits a significant PPR lasting over four minutes when the arm is immersed almost up to the shoulder for two minutes (8). We hypothesize that multiple factors could explain these results, such as the intensity of pain perception, its unpleasantness, as well as the spatial and temporal summation elicited by CPT.

While CPT has recently been identified as being able to trigger PPR, traditionally, this test was used to study inhibitory conditioned pain modulation (ICPM). Indeed, ICPM is one of the principal endogenous pain inhibition mechanisms whereby a nociceptive stimulation, or conditioning stimulus, applied remotely, will produce a diffuse analgesic effect (8, 23–26). For this to occur, the conditioning stimulus, in this case the CPT, must be administered for a relatively prolonged time on a large body surface (e.g., the forearm). To measure the ICPM effects, a sequential experimental design, inducing moderate pain with a thermode, has often been used (8, 27). The heating plate was set to an individualized temperature and applied for two-minute periods on the left forearm of participants before and after CPT which also lasted two minutes. This experimental design has repeatedly demonstrated a 20%–30% reduction in pain, and a decrease in ICPM efficacy in certain clinical populations including people suffering from fibromyalgia and irritable bowel syndrome (27–29).

Despite CPT showing overall robust effects (PPR & ICPM), they vary greatly from one person to another (8, 30, 31). However, research on interindividual variability regarding PPR is sparse. A preliminary study noted significant correlations between the pain intensity and unpleasantness perceived during CPT and the magnitude of PPR felt by participants (8). In the case of ICPM, more research has been conducted on interindividual variability. In general, it has shown a positive relationship between efficacy of ICPM, and the intensity and unpleasantness of pain perceived during the conditioning stimulus (32–34). Nonetheless, not all studies have obtained such relationships (35, 36). Furthermore, ICPM seems to be more efficient in young people and in men (36–39). Relationships with psychological variables (e.g., pain catastrophizing, depression, and anxiety) have also been observed, but the strength of these associations remains rather small (40, 41). One fundamental limit of the studies carried out on interindividual variability is the use, in most cases, of correlational statistical analyses based on mean pain scores, when tonic nociceptive stimuli are used. These methods are limited as they forgo the exploitation of rich dynamic pain responses throughout noxious stimulation (33, 42). Yet, some studies suggest that the amplitude of ICPM could be influenced by temporal summation effects occurring during the administration of the conditioning stimulus (43, 44).

This data driven study seeks to identify clusters of participants displaying similar dynamic pain responses during a tonic conditioning stimulus and investigate factors of variability for PPR and ICPM between healthy volunteers. While PPR (a compensatory pleasant experience) and ICPM (endogenous analgesia) can both be triggered by the CPT, the levels of PPR and ICPM do not necessarily correlate (8). To identify clusters, dynamic variations of participants’ pain responses over two minutes during the administration of CPT will be exploited. More specifically, we will perform trajectory analyses using the pain intensity and unpleasantness scores collected five times during the conditioning stimulus. Once distinct groups have been identified, we will verify if the degree of PPR as well as the efficacy of the ICPM are different between trajectories. Considering that the current study involves healthy volunteers, we expect clusters of participants, characterized by a stronger response to pain or a higher degree of sensitization during CPT, to have a greater PPR and ICPM efficacy.

## Materials and methods

### Participants

One hundred and twenty-two (122) healthy volunteers between 18 and 35 years old were recruited for this study [80 women and 42 men; mean age 24.2 ± 4.7; mean ± standard deviation (SD)] (Table 1). Participants that corresponded to any of the following criteria were excluded: (1) any DSM-V axis psychiatric disorder (including substance use disorders), (2) centrally acting medication, (3) neurologic disorders, (4) any unstable medical condition, and (5) history of chronic pain. None of the participants suffered from chronic pain or had significant acute painful symptoms as determined with the *Brief Pain Inventory* (BPI; mean pain 0.7 ± 0.1) (50, 51). Subclinical psychological symptoms of depression, anxiety and anhedonia were evaluated, respectively, with the *Beck Depression Inventory-II* (BDI-II) (52), the *State and Trait Anxiety Inventory*-*state* subscale (STAI-S) (53, 54), and the *Snaith-Hamilton Pleasure Scale* (SHPS) (47, 48). Recruitment was done by word of mouth and through online advertisements (Kijiji, Facebook). Each participant signed a detailed consent form in accordance with the 1,964 Declaration of Helsinki, and the local ethics committee approved the research.

**Table 1:** Psychosocial characteristics of participants.

Characteristics	Statistics
Age (mean ± SEM)	24.2 ± 0.4
Sex (%)	
Male	34.5
Female	65.5
Ethnicity (%)	
Caucasian	50.8
Black or African American	16.4
Latin American	6.6
Asian	11.5
Other	8.2
Multiethnic	6.6
Years of education (mean ± SEM)	16.1 ± 0.02
Employment status (%)	
Employed	53.3
Unemployed	30.3
Other	17.2
Psychological symptoms (M ± SEM)	
BDI-II^a^	6.5 ± 0.5
STAI-S^b^	31.9 ± 0.7
SHPS^c^	0.6 ± 0.1
BPI (mean ± SEM)	
Pain severity	1.6 ± 0.4
Pain interference	1.2 ± 0.5

BDI-II, Beck Depression Inventory; M, mean; SEM, standard error of the mean; SHPS, Snaith–Hamilton Pleasure Scale; STAI, State and Trait Inventory.

^a^ No depression (0–13) (45).

^b^ No or low anxiety (20–37) (46).

^c^ Normal hedonia (0–3) (47–49).

### Conditioning stimulus for PPR

CPT consisted of the immersion of the right arm up to the shoulder, for a maximum of two minutes, into a bath of cold water. A refrigerated circulation system (Julabo F33-HL heating/refrigerating circulators) kept the water at a constant 10°C throughout the experiment. The temperature was chosen to be painful yet tolerable for two minutes (27). During the administration of the conditioning stimulus, participants were instructed to report verbally pain intensity and pain unpleasantness on a scale of 0 (no pain) to 100 (worst pain imaginable/most unpleasant imaginable) at the beginning, then at 30-second intervals up to 120 s. Throughout the manuscript, we refer to this conditioning stimulus as CPT-PPR.

#### Pleasant pain relief

To illustrate the PPR phenomenon, we provided an example like the one used in a previous study (9). Participants were asked to imagine themselves walking in a −30°C snowstorm for 20 min and finally arriving home to feel the warmth of the air inside the house. This heat would induce feelings of both relief and pleasure (9) elicited by the cessation of the painful stimulus. To assess the PPR, participants were asked to rate it on a scale of 0 [“I feel relief, but no pleasure”] to 100 [“I feel relief and the most intense pleasure possible”]. PPR was measured immediately after the end of the immersion and every 30 s afterwards for four minutes. These ratings were used to calculate the mean, the first (score at CPT offset) PPR and the peak (highest score) PPR of each participant.

### Inhibitory conditioned pain modulation paradigm

**Figure 1:**
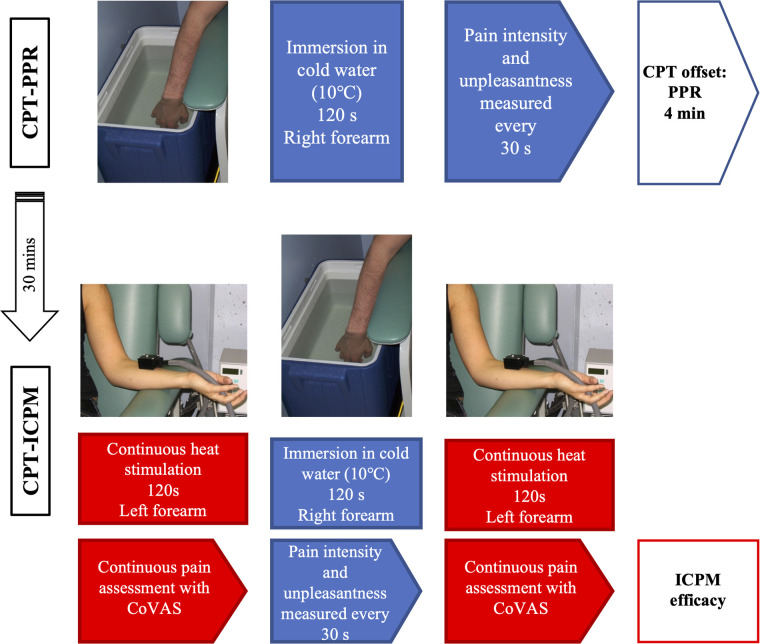
Schema of the CPT-PPR and CPT-ICPM protocols.

The inhibitory CPM paradigm was administered 30 min apart from the CPT-PPR to prevent pain sensitization. The order of CPT-PPR and CPT-ICPM was not counterbalanced between subjects (Figure 1).

#### Heat pain threshold and tolerance

Thermal pain threshold and tolerance were measured by applying a 3 cm^2^ Peltier thermode on the left forearm of participants (TSA-II, Medoc, Advanced Medical Systems, Ramat Yishai, Israel) (27). This heating plate was connected to a computer and allowed precise control of temperatures. Experimental temperatures started at 32°C and gradually increased at 0.3°C per second. Participants were asked to report the moment at which the heat started to be painful (thermal pain threshold), and when pain became intolerable (pain tolerance threshold) (9, 27). For each participant, the temperature inducing moderate pain (T50) was also measured. Upon the first application (pretest 1), these measures were taken verbally to ensure the participant’s understanding of the procedure. During the second and third application (pretests 2 and 3), these measures were reported by participants using a computerized visual analog scale (CoVAS). This scale ranged from 0 (no pain) to 100 (most intense pain tolerable) (27). Hence, when the pain started (CoVAS = 1) they moved the scale’s cursor until pain perception reached its maximum (CoVAS = 100), at which point the heating of the thermode was immediately stopped and brought back to a non-painful temperature. To ensure that there would be no peripheral sensitization, the placement of the thermode was moved between each stimulation (distal to proximal) for all subjects.

#### Tonic heat pain perception

The test stimulus consisted of a continuous heat stimulation inducing moderate pain (T50) for two minutes (27). This heat stimulation was administered with a thermode on the left forearm of participants. The temperature of the thermode started at 32°C and increased at 0.3°C/sec until it reached an individually predetermined T50 (based on heat pain threshold and tolerance). It then remained constant at this temperature for two minutes. However, participants were told that the temperature was randomly fluctuating, in a range that they would be able to tolerate (55). During the administration of the test stimulus, individuals were instructed to measure pain intensity using the same CoVAS previously described (continuous ratings with pain intensity measured at a 1-second rate). This test was administered twice: before and after the CPT-ICPM. We compared the pain ratings of the test stimulus before and after the conditioning stimulus as an index of ICPM efficacy (pain inhibition in percentage).

#### Condition stimulus for the elicitation of inhibitory CPM

To induce the ICPM with the CPT, we re-administered this conditioning stimulus with the same parameters as the CPT-PPR (section 2.2.). This test will be referred to as CPT-ICPM.

## Statistical analyses

We used group-based trajectory modelling (GBTM) analyses to categorize the variations in CPT pain intensity and unpleasantness scores over time using a SAS procedure PROC TRAJ (56). The number of trajectories was determined based on the statistical model fit criteria, the class size (at least 5% of participants), and the interpretation of the classes (57, 58). The statistical criteria examined were the Bayesian information criteria (BIC), the odds of correct classification, and the average posterior probability (57, 59, 60). The model that minimized the absolute value of the Bayesian information criteria was determined to be the best fit statistically (59). Also, Nagin et al. recommended that the trajectories had a group membership over 5% (group percent estimates, $\hat{\pi }j$), an average posterior probability above 0.7 and, a minimum odd of correct classification of 5 for all groups (60). The optimal number of classes was identified by analyzing 1-class through to 6-class models, with several polynomial types (linear, quadratic, and cubic) (61). The censored normal (CNORM) model was used, and a GBTM was computed for pain intensity and pain unpleasantness during CPT-PPR, as well as pain intensity and pain unpleasantness during the CPT-ICPM. Then, we performed analyses of variance (ANOVA) to identify potential differences between the subgroups obtained using the trajectory analyses and the PPR scores and ICPM efficacy, as primary outcomes. In the case of CPT-PPR, the first and peak PPR scores were also considered as secondary outcomes. We also performed two sets of analyses, based on CPT intensity and unpleasantness scores, between CPT subgroups. Paired t-tests were performed to compare pain ratings before and after CPT-ICPM for ICPM efficacy. To make sure that pleasant-pain results are not confounded by socio-demographic (age and sex) and psychological variables (BDI, BPI, STAI and SHPS), ANOVAs were performed for continuous variables (age & psychological scales), and chi-square analyzes for dichotomous variables (sex) using SPSS, version 27. All results are presented as mean ± standard error of the mean (SEM). Results were considered significant at *p* < 0.025 (0.05 divided by 2 sub-analyses on intensity and unpleasantness), and a Tukey correction was applied for multiple comparisons between sub-groups.

## Results

### Pleasant pain relief

**Figure 2:**
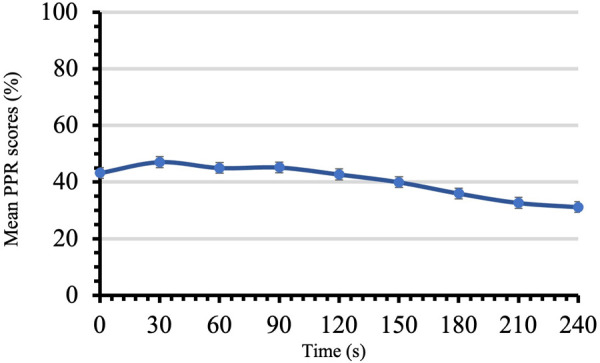
PPR scores in time for CPT-PPR. Mean PPR scores on a scale from 0 to 100 are reported for four minutes during the administration of the conditioning stimulus for PPR. Each time point shows mean ± SEM.

After the CPT-PPR, PPR measures were taken every 30 s for four minutes (Figure 2). The mean PPR was 41.2 ± 2.7, and the first and peak PPR by participant were 43.2 ± 2.7 and 63.4 ± 2.6 respectively.

#### Cold pressor test (PPR) and group-based trajectory modelling

**Figure 3:**
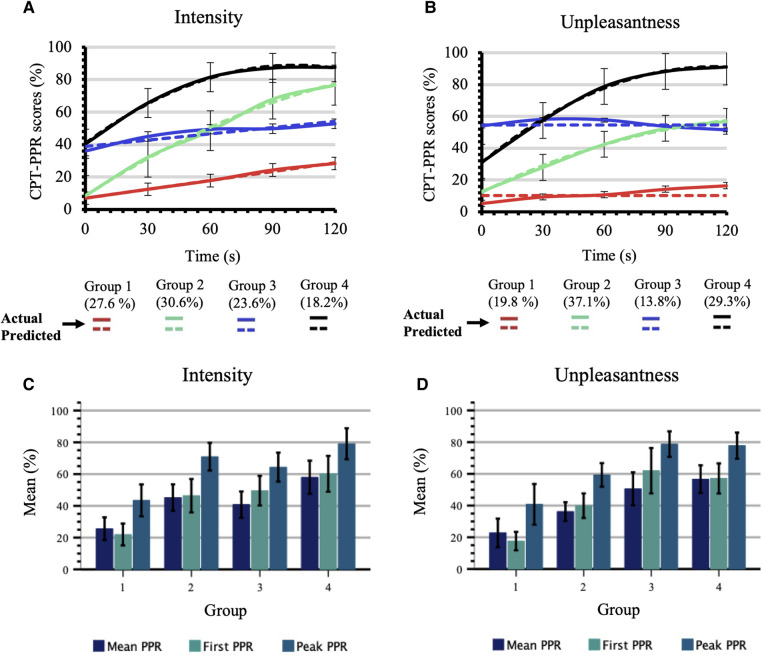
CPT-PPR trajectories and related PPR scores across groups. (**A**) CPT-PPR during 120 s by group trajectories for pain intensity scores. Each time point shows mean ± SEM. (**B**) CPT-PPR during 120 s by group trajectories for pain unpleasantness scores. Each time point shows mean ± SEM. (**C**) Scores for mean PPR, peak PPR, and first PPR for the CPT-PPR subdivided by groups obtained in A. Each bar shows mean ± 95% CI. (**D**) Scores for mean PPR, peak PPR, and first PPR for the CPT-PPR subdivided by groups obtained in B. Each bar shows mean ± 95% CI. All values are scored on a 0 to 100 scale.

During the administration of CPT-PPR, mean pain intensity and unpleasantness scores were, respectively, 42.8 ± 1.7, and 43.6 ± 2.0. Four trajectories were obtained from trajectory modelling analyses for pain intensity and pain unpleasantness during CPT-PPR: low sensitivity-low increasing pain perception (group 1), low sensitivity-high increasing pain perception (group 2) moderate sensitivity-steady pain perception (group 3) and moderate sensitivity- moderate increasing pain perception (group 4) (Figures 3A,B); please refer to Supplementary Table S1 for model selection criteria. The comparison of intensity and unpleasantness group membership produces a kappa value of 0.42 (*p* < 0.001) which suggests a moderate strength of agreement between trajectories (62).

#### Psychophysical measures and cold pressor test intensity trajectories

Mean pain intensity measures varied between CPT-PPR defined groups. As seen in Table 2, group 1 had a mean of 17.9 ± 1.3, group 2, of 46.7 ± 1.3, group 3, of 46.2 ± 1.4, and group 4 of 68.3 ± 1.6. These results were significantly different between all groups (*p* < 0.001) except for groups 2 and 3 for which results did not differ (*p* = 0.995). Concerning psychological measures, a difference was observed between CPT-PPR trajectories and their mean PPR [F(3,118) = 9.736, *p* < 0.001], first PPR [F(3, 118) = 11.603, *p* < 0.001] and peak PPR scores [F(3,118) = 10.591, *p* < 0.001] (Table 2 and Figure 3C). More specifically, for the mean PPR, group 1 had lower scores than all other groups as group 3 which had less PPR than group 4. There were also significant differences in first and peak PPR when comparing group 1 to the three other groups. Indeed, group 1 had lower peak PPR when compared to groups 2, 3 and 4 (Table 2). There were no differences across trajectories for sex [F(3,118) = 0.777, *p* = 0.509] and age [F(3,118) = 1.303, *p* = 0.277]. Finally, scores on psychological questionnaires did not differ across groups: BDI-II [F(3,118) = 0.170, *p* = 0.916], SHPS [F(3,118) = 0.142, *p* = 0.935], STAI-S [F(3,118) = 0.395, *p* = 0.757] and BPI intensity and interference scores [resp. F(3,118)= 0.411, *p* = 0.745 and F(3,118) = 1.871, *p* = 0.138].

**Table 2:** PPR scores for CPT-PPR intensity and unpleasantness trajectories.

CPT-defined groups (scores = M ± SEM)	Mean PPR (M ± SEM)	First PPR (M ± SEM)	Peak PPR (M ± SEM)
Intensity			
Gr 1 (17.9 ± 1.3)	25.7 ± 3.5	22.0 ± 3.4	43.5 ± 4.9
Gr 2 (46.7 ± 1.3)	45.3 ± 4.1	46.4 ± 5.2	71.0 ± 4.3
Gr 3 (46.2 ± 1.4)	40.8 ± 4.1	49.6 ± 4.6	64.4 ± 4.5
Gr 4 (68.3 ± 1.6)	58.0 ± 5.0	60.2 ± 5.4	79.1 ± 4.7
Significant differences	Gr 1 < Gr 2**, 3* & 4*** Gr 3 < Gr 4*	Gr 1 < Gr 2/3/4***	Gr 1 < Gr 3** & 2 /4***
Unpleasantness			
Gr 1 (11.5 ± 1.3)	22.8 ± 4.3	17.7 ± 2.8	40.8 ± 6.2
Gr 2 (38.5 ± 1.4)	36.3 ± 2.9	40.0 ± 3.9	59.4 ± 3.7
Gr 3 (55.0 ± 2.3)	50.6 ± 4.8	62.0 ± 6.7	78.8 ± 3.7
Gr 4 (67.7 ± 1.8)	56.7 ± 4.3	57.1 ± 4.7	77.8 ± 4.1
Significant differences	Gr 1 < Gr 3** & 4*** Gr 2 < Gr 4***	Gr 1 < Gr 2** & 3/4*** Gr 2 < Gr 3*** & 4*	Gr 1 < Gr 2* & 3/4*** Gr 2 < Gr 3* & 4**

Gr, group; M, Mean; SEM, standard error of the mean.

* *p* < 0.05; **p < 0.01; ***p < 0.001.

#### Psychophysical measures and cold pressor test unpleasantness trajectories

Pain unpleasantness measures varied between CPT-PPR-defined groups. When compared, all groups had significantly different mean unpleasantness scores [F(3,118) = 189.214 *p* < 0.001]: group 1 = 11.5 ± 1.3; group 2, 38.5 ± 1.4; group 3, 55.0 ± 2.3; and group 4, 67.7 ± 1.8 (Table 2). Moreover, a significant difference was observed between the CPT-PPR trajectories and their mean PPR [F(3,118) = 13.115, *p* < 0.001], first PPR [F(3,118) = 15.172, *p* < 0.001] and peak PPR scores [F(3,118) = 12.606, *p* < 0.001] (Table 2 and Figure 3D). For the mean PPR, when we looked at group pairings, we found that these differences resided between group 1 and groups 3 and 4, as well as between groups 2 and 4. For first and peak PPR, significant differences were observable between group 1 and all other groups; also, group 2 had lower PPR ratings than groups 3 and 4 (Table 2). In the comparisons for mean, first and peak PPR, groups 1 and 2 displayed lower PPR scores than their counterparts. No significant differences were found between groups for sex and age [resp. F(3,118) = 0.273, *p* = 0.845 and F(3,118) = 0.337, *p* = 0.799]. Finally, no significant differences were obtained between groups for BDI-II [F(3,118) = 0.534, *p* = 0.660], SHPS [F(3,118) = 0.163, *p* = 0.921], STAI-S [F(3,118) = 0.516, *p* = 0.672] and BPI intensity and interference scores [resp. F(3,118) = 2.399, *p* = 0.071 and F(3,118) = 0.895, *p* = 0.446]).

### Inhibitory conditioned pain modulation paradigm

#### Heat pain threshold and tolerance

During the pretest, the thermal pain threshold of participants was 40.7 ± 0.4°C, the thermal pain tolerance was 47.2 ± 0.2°C, and the T50 was 45.2 ± 0.2°C.

#### ICPM

**Figure 4:**
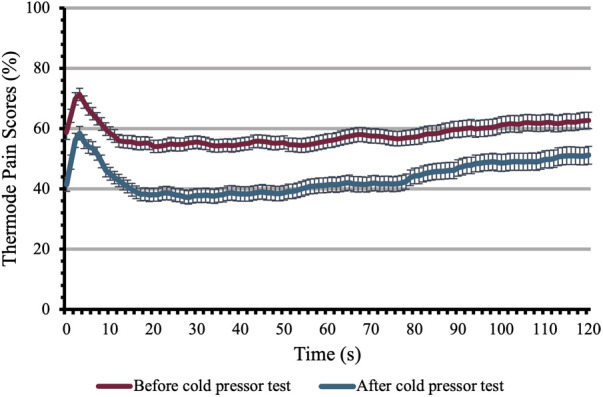
Inhibitory conditioned pain modulation. This figure shows the pain perception of participants to the heating thermode before and after the conditioning stimulus. Pain perception was evaluated during both tests on a scale of 0 to 100 for 120 s. Each time point shows the mean ± SEM.

During the CPT-ICPM, the mean pain intensity and mean pain unpleasantness were, respectively, 58.2 ± 1.8 and 49.3 ± 2.7. The mean pain ratings for the test stimulus administered before the conditioning stimulus were 58.1 ± 1.9 and were reduced to 43.7 ± 2.1 after the conditioning stimulus (mean difference 14.4 ± 1.8) which represents a mean inhibition of 23.3 ± 3.13% (Figure 4). The difference between these pain ratings was significant [t(121) = 7.878; *p* < 0.001].

#### CPT-ICPM trajectories and ICPM efficacy

Four trajectories were obtained for pain intensity and pain unpleasantness trajectory modelling analyses during the CPT-ICPM. The trajectories were similar to those of CPT-PPR and included the following groups: low sensitivity-low increasing pain perception (group 1), low sensitivity-high increasing pain perception (group 2) moderate sensitivity-steady pain perception (group 3) and moderate sensitivity- moderate increasing pain perception (group 4). However, no differences were significant between the CPT-ICPM pain intensity or pain unpleasantness trajectories and mean ICPM efficacy [resp. F(3,118) = 0.982, *p* = 0.404 and F(3,118) = 0.936, *p* = 0.426] (see Supplementary Figure S1 and Tables S2, S3). The level of agreement between CPT-PPR and CPT-ICPM for intensity and unpleasantness produced a moderate kappa value of 0.48 and 0.52 respectively (*p* < 0.001 for both measures). Sociodemographic variables and psychological symptoms showed no differences between groups (*p* > 0.05).

## Discussion

The goal of the present study was to distinguish groups which had similar CPT intensity and unpleasantness responses over time, using a robust data-driven approach. Once identified, differences between those groups, regarding PPR and ICPM efficacy, were evaluated. ICPM has been extensively studied over the years and CPT has proven to be a reliable and effective method to induce such phenomenon (19, 31, 63). In addition to inducing analgesia, it appears that the offset of a conditioning stimulus induces a pleasant relief. While ICPM and PPR are well described phenomena, analyses on interindividual variability in PPR has been rarely examined (5). In this study, GBTM analyses revealed the presence of four distinct trajectories based on both CPT intensity and unpleasantness scores measured five times during the two-minute pain administration. Mean PPR, first PPR and peak PPR were significatively different between the identified groups. PPR increased with CPT pain intensity and unpleasantness scores -in other words, participants with greater pain perception during CPT, felt a more pleasurable relief at its offset. However, while PPR levels were influenced by both global pain intensity and pain intensity sensitization during the CPT, ICPM efficacy was only influenced by the global pain unpleasantness experienced during the CPT (as discussed below). Consistent with the results of previous studies, CPT-ICPM induced a significant and robust reduction in pain perception (31, 63). Furthermore, contrary to findings indicating that ICPM is positively correlated to pain perception during the conditioning stimulus, no differences were shown when comparing the degree of ICPM efficacy between CPT-ICPM trajectories (9, 34, 64). The differences between CPT-ICPM and CPT-PPR might be driven by the distinct systems that mediate them. Indeed, ICPM recruits structures in the brainstem while PPR is mostly modulated by activations in the reward system (12, 13, 23, 24). However, the use of group-based trajectory analyses yielded interesting results, that complement previous findings, and enabled the investigation of unexplored territories in research on PPR and ICPM mechanisms. Hence, this method bears promising grounds for future studies on pain modulation.

In accordance with previous studies, PPR scores were positively correlated with pain ratings (both intensity and unpleasantness) during CPT (8, 9, 65). Even for groups reporting lower levels of pain intensity or unpleasantness during CPT, a PPR was elicited. However, it appears that when a certain threshold is reached, PPR levels significantly increase. Indeed, group 1 (“low pain perception”; i.e., <30/100 throughout the CPT) had systematically lower PPR when compared to the three other groups. This was true for both pain intensity and pain unpleasantness during the CPT. Perhaps more importantly, separating pain dimensions (intensity and unpleasantness) revealed that their trajectories, compared to mean values may have distinct effects on PPR ratings, especially when comparing groups 2, 3 and 4. Interestingly, group 4 reported higher pain intensity during CPT than group 2, but its PPR scores were not significantly higher. Moreover, groups 2 and 3 had very similar mean pain intensity ratings, which were both significantly different from those of group 4. Yet only group 3 had significantly lower mean PPR than group 4, unlike group 2, which had similar PPR ratings compared to group 4. Therefore, the fact that group 2 displayed more PPR than group 3 might be linked to its pain intensity being higher at 120 s relatively to group 3. Consequently, PPR scores could be more related to the pain intensity reported at the end of the two minutes, instead of its mean score, indicating a positive relationship between this variable and temporal summation (e.g., slope) effects of CPT (e.g., pain sensitization).

The opposite phenomenon is present for pain unpleasantness. Although unpleasantness scores were similar for groups 2 and 3 at the end of CPT-PPR (120 s), it was group 3, reporting higher mean pain unpleasantness, that showed a more intense PPR, suggesting that it is the pain unpleasantness felt throughout the entire CPT that has the most impact. Undoubtedly, the use of a data-driven approach based on dynamic pain responses has allowed us to study PPR inter-subject variability in a manner that would have not been feasible had we relied on mean pain experience during CPT and basic correlational analyses only. Considering the novelty of the current findings, the precise neurophysiological mechanisms involved in the subtle differences in the relationship between PPR, pain intensity and pain unpleasantness remain elusive, and merit future investigation.

Another goal of this study was to evaluate the potential relationships between ICPM efficacy and CPT-ICPM trajectories. While CPT produced a robust ICPM, the level of pain intensity or unpleasantness reported during the conditioning stimulus did not appear to have an impact on the degree of pain inhibition between groups. These results are in contradiction with previous studies on healthy individuals showing that ICPM efficacy is positively correlated to CPT pain intensity and unpleasantness (66–68). It is important to point out that findings regarding the relationship between pain intensity/unpleasantness during the conditioning stimulus and ICPM efficacy are inconsistent, as some of them reported no significant correlation between these variables (8, 35, 69, 70). Many factors could explain these discrepancies, such as the intensity (e.g., temperature of the CPT) and duration of the conditioning stimulus (34), its modality (e.g., thermal vs. ischemic), the paradigm used (parallel vs. sequential) (32, 42, 64), socio-demographic variables, including sex (39) and age (38) as well as psychological factors (e.g., pain catastrophizing) (41). In this study, socio-demographic and psychological variables were thoroughly assessed, and no association between these variables and the CPT-PPR and CPT-ICPM groups were found. Furthermore, it is relevant to mention that some evidence suggests that mild or even non-painful conditioning stimulus could also induce ICPM (71, 72). Although most studies show that a painful stimulation is required to induce ICPM, some studies indeed suggest that a strong and long-lasting but non-painful stimulus can also trigger pain inhibition. Since subjective pain ratings do not always correlate with ICPM efficacy, it has been argued that activation of ICPM has physiological roots, requiring only a certain threshold of stimulation coming from peripheral nociceptors to be triggered (73).

This article has a few limitations. First, the sample size is relatively small especially for GBTM. The creation of clusters reduces the number of participants per group and can impact statistical power. Despite this, robust trajectories were identified using stringent selection criteria, which allowed the detection of significant variability between groups, including subtle differences between similar trajectories such as groups 3 and 4 identified in the intensity analyses of CPT-PPR data (58, 60, 74, 75). Second, even if a sequential protocol for the administrations of the test stimuli (e.g., thermode) and the conditioning stimulus (e.g., CPT) is recommended by some authors, as it limits biases due to distraction, a parallel paradigm has been shown to induce a more pronounced ICPM mostly because pain inhibition, at noxious stimulation offset, is time sensitive (31, 76). However, a recent study found no differences between sequential and parallel test designs in terms of ICPM intensity (77). It remains to be determined if the use of a parallel paradigm would have enabled the detection of a correlation between CPT groups and ICPM efficacy.

This study has provided a first step into the investigation of PPR and ICPM efficacy interindividual variability. Using a data-driven approach provided more in-depth information on such mechanisms than traditional correlational analyses. It was shown that PPR at CPT offset differs between clusters of participants identified based on dynamic pain intensity and unpleasantness responses from CPT. Thus, it was brought to light that both pain experience and pain sensitization (e.g., temporal effects) may underlie individual differences in PPR responses. Such findings would not have been possible without the clustering of similar pain experience trajectories during the CPT. Future investigations using similar analyses should be done on larger samples of participants to provide further knowledge on the links between trajectories and PPR. Such data could contribute to a deeper understanding of the mechanisms influencing the interindividual variability of pain modulation. Moreover, it would be highly relevant to examine these complex relationships in patients suffering from chronic pain conditions and/or substance use disorders known to be associated with altered pain modulation mechanisms. Considering that high levels of pain sensitization are observed in chronic pain patients, the patterns observed here in healthy controls might significantly differ in clinical populations. In that endeavor, it would be of interest to perform head-to-head comparisons of the predictive value of data-driven vs. traditional approaches.

## Data Availability

The data that support the findings of this study are available upon reasonable request from the corresponding author SP but are only redistributable to researchers engaged in IRB approved research collaborations.
