# Supplementary material for: Inter-subject variability of pleasant pain relief using a data-driven approach in healthy volunteers
Source: Front Pain Res (Lausanne). 2022 Nov 21;3:1003237. doi: 10.3389/fpain.2022.1003237 (PMC9720129; doi:10.3389/fpain.2022.1003237)
Supplement: Supplementary file 1 [file Datasheet1.docx]

Supplementary Material


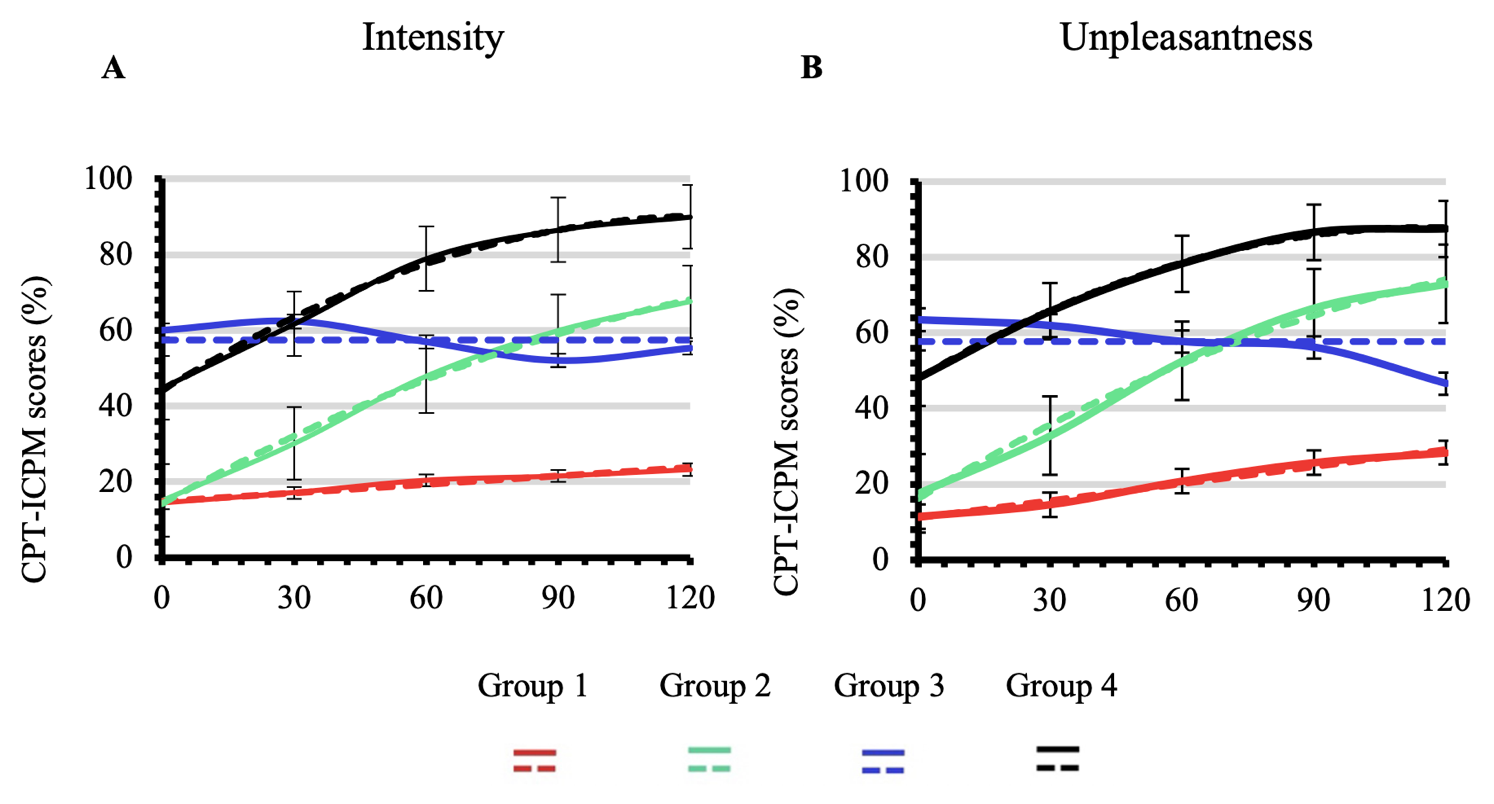


**Supplementary Figure 1**. CPT-ICPM Intensity and Unpleasantness Trajectories. (A) CPT-ICPM intensity scores during 120 seconds by group trajectories. (B) CPT-ICPM unpleasantness scores during 120 seconds by group trajectories. The level of agreement between intensity and unpleasantness group membership is $\kappa$=0.51 (p<0.001). Each time point shows mean ± SEM.

**Supplementary Table 1:** Criteria for Model Selection and Adequacy of CPT-PPR Trajectories

| **Group** | n | AvePPj | $\hat{\pi}$j | OCC |
| --- | --- | --- | --- | --- |
| **1** | 33 | 99.2% | 27.6% | 306.4 |
| **2** | 35 | 93.4% | 30.6% | 32.0 |
| **3** | 31 | 85.2% | 23.6% | 18.7 |
| **4** | 23 | 93.8% | 18.2% | 67.8 |

| Model/# of groups | BIC |
| --- | --- |
| 1 | -2592 |
| 2 | -2491 |
| 3 | -2440 |
| **4**  Intensity | **-2428** |
| 5 | -2408 |
| 6 | -2421 |

| Model/# of groups | BIC |
| --- | --- |
| 1 | -2576 |
| 2 | -2444 |
| 3 | -2399 |
| **4** | -**2385** |
| 5 | -2396 |
| 6 | -2335 |

| **Group** | n | AvePPj | $\hat{\pi}$j | OCC |
| --- | --- | --- | --- | --- |
| **1** | 24 | 91.9% | 19.8% | 45.8 |
| **2** | 48 | 93.3% | 37.1% | 23.5 |
| **3** | 15 | 98.6% | 13.8% | 446.1 |
| **4** | 35 | 93.2% | 29.3% | 33.0 |

Unpleasantness

AvePPj, Average posterior probability; BIC**,** Bayesian information criteria; OCC, Odds of correct classification,$\hat{\pi}$j, Group percent estimates.

**Supplementary Table 2:** Criteria for Model Selection and Adequacy of CPT-ICPM Trajectories

| Model/# of groups | BIC |
| --- | --- |
| 1 | -2594 |
| 2 | -2505 |
| 3 | -2491 |
| **4** | **-2449** |
| 5 | -2433 |
| 6 | -2411 |

| **Group** | n | AvePPj | $\hat{\pi}$j | OCC |
| --- | --- | --- | --- | --- |
| **1** | 23 | 93.4% | 18.3% | 63.7 |
| **2** | 54 | 95.2% | 44.6% | 24.8 |
| **3** | 15 | 97.2% | 13.8% | 217.9 |
| **4** | 29 | 93.8% | 23.3% | 49.9 |

Intensity

| **Group** | n | AvePPj | $\hat{\pi}$j | OCC |
| --- | --- | --- | --- | --- |
| **1** | 36 | 97.1% | 29.5% | 77.9 |
| **2** | 41 | 88.9% | 32.0% | 19.1 |
| **3** | 13 | 91.1% | 14.0% | 28.9 |
| **4** | 31 | 89.3% | 24.6% | 50.3 |

| Model/# of groups | BIC |
| --- | --- |
| 1 | -2596 |
| 2 | -2461 |
| 3 | -2450 |
| **4** | **-2418** |
| 5 | -2419 |
| 6 | -2398 |

Unpleasantness

AvePPj, Average posterior probability; BIC**,** Bayesian information criteria; OCC, Odds of correct classification, j, Group percent estimates.

**Supplementary Table 3:** ICPM Scores for CPT-ICPM Intensity and Unpleasantness Trajectories

| **CPT-defined groups**  (scores = M ± SEM) | **ICPM efficacy**  (M ± SEM) |
| --- | --- |
| Intensity |  |
| Gr 1 (19.3 ± 2.1) | 14.6 ± 7.9 |
| Gr 2 (43.5 ± 1.1) | 28.7 ± 4.6 |
| Gr 3 (56.7 ± 1.9) | 19.2 ± 9.9 |
| Gr 4 (70.6 ± 1.5) | 26.3 ± 3.2 |
| Statistics | F(3)=0.982, p=0.404 |
| Unpleasantness |  |
| Gr 1 (20.2± 1.6) | 16.1 ± 6.3 |
| Gr 2 (48.5 ± 1.4) | 27.8 ± 5.6 |
| Gr 3 (57.2 ± 3.5) | 25.9 ± 9.3 |
| Gr 4 (73.2 ± 1.3) | 28.5 ± 5.9 |
| Statistics | F(3)=0.936 p=0.426 |

Gr, group; M, Mean; SEM, standard error of the mean.
